# Modulation of Mu-Opioid Receptor Expression and Functional Impairment of Natural Killer Cells in Neuropathic Pain: Implications for Biomarker Discovery and Personalized Therapies

**DOI:** 10.3390/ph19060933

**Published:** 2026-06-13

**Authors:** Lucia Carmela Passacatini, Saverio Nucera, Rosamaria Caminiti, Valentina Malafoglia, Valeria Mazza, Leonardo Lupacchini, Stefania Proietti, Laura Vitiello, Roberta Macrì, Maria Serra, Francesca Oppedisano, Jessica Maiuolo, Cinzia Garofalo, Carlo Tomino, Vincenzo Mollace, Sara Ilari, William Raffaeli, Carolina Muscoli

**Affiliations:** 1 Pain Physiology and Pharmacology, IRCCS San Raffaele Roma, 00166 Rome, Italy; valentina.malafoglia@sanraffaele.it; 2 Department of Health Sciences, Institute of Research for Food Safety and Health (IRC-FSH), University “Magna Græcia” of Catanzaro, 88100 Catanzaro, Italy; saverio.nucera@unicz.it (S.N.); foppedisano@unicz.it (F.O.);; 3 Department of Medical and Clinical Surgery, University “Magna Græcia” of Catanzaro, 88100 Catanzaro, Italy; 4 Laboratory of Molecular and Cellular Neurobiology, IRCCS San Raffaele Roma, 00166 Rome, Italy; leonardo.lupacchini@sanraffaele.it; 5 Agea, Coordinating Body, 00185 Rome, Italy; 6 Department of Human Sciences and Quality of Life Promotion, San Raffaele University, 00166 Rome, Italy; laura.vitiello@uniroma5.it; 7 Clinical and Molecular Epidemiology, IRCCS San Raffaele Roma, 00166 Rome, Italy; 8 Department of Experimental and Clinical Medicine, University “Magna Græcia” of Catanzaro, 88100 Catanzaro, Italy; cinziagarofalo@unicz.it; 9 IRCCS San Raffaele Roma, 00166 Rome, Italy; 10 ISAL Foundation, Institute for Research on Pain, 47921 Rimini, Italy

**Keywords:** chronic neuropathic pain, chronic constriction injury, μ-opioid receptor, natural killer cells, degranulation, pain biomarkers, chronic pain biomarkers

## Abstract

**Background/Objectives**: Chronic pain is a significant clinical challenge, in part due to the absence of reliable objective biomarkers for its evaluation and treatment. Growing evidence indicates that immune cells, including natural killer (NK) cells, are involved in the regulation of pain processes. NK cells are innate cytotoxic lymphocytes whose functional status may mirror underlying pathological pain states. In this study, we investigated μ-opioid receptor (MOR) expression and functional alterations of NK cells in a murine model of neuropathic pain induced by chronic constriction injury (CCI). **Methods**: Mice were divided into three groups: Sham (sciatic nerve exposure without ligation), CCI 14-day, and CCI 21-day groups. At the respective time points, animals were sacrificed and spleens were collected for analysis. Splenocytes were isolated by mechanical dissociation followed by centrifugation and erythrocyte lysis. Lymphocytes were analyzed by flow cytometry to evaluate MOR expression in NK cells and their degranulation activity (CD107a assay). Cells were incubated with fluorochrome-conjugated antibodies against NK cell markers (NK1.1, CD3, Ly49A, Ly49C/I) in combination with anti-MOR and anti-Interferon γ antibody (IFN-γ). Immunofluorescence and confocal microscopy analyses were performed to assess MOR localization and granzyme localization, supporting CD107a-mediated degranulation. **Results**: Flow cytometry analysis revealed a significant reduction in surface MOR expression on total NK cells from CCI mice compared with sham controls at 14 and 21 days post-injury, a finding corroborated by immunofluorescence evidence of MOR cellular internalization. Functionally, CCI induced a marked decrease in CD107a expression and impaired IFN-γ production both under basal conditions and following PMA/ionomycin stimulation, indicating a hyporesponsive state of NK cells. Consistently, confocal microscopy revealed extracellular release of Granzyme A following CCI, suggesting dysregulated degranulation. **Conclusions**: Neuropathic pain is associated with a remodeling of NK cell phenotype and effector functions, characterized by impaired cytotoxic activity and cytokine production, along with modulation of inhibitory receptor expression. Notably, MOR-reduced surface expression in NK cells emerges as a potential biomarker of neuropathic pain. Further studies are needed to elucidate the molecular mechanisms regulating MOR expression and its relationship with NK cell hyporesponsiveness and degranulation in chronic pain conditions.

## 1. Introduction

Pain represents a major clinical and public health issue worldwide [1]. Chronic pain, in particular, constitutes a major social, clinical, and economic burden, severely affecting patients’ quality of life and representing a persistent challenge for healthcare and clinicians [2].

Among chronic pain conditions, neuropathic pain, caused by lesion or dysfunction of the somatosensory nervous system, remains one of the most difficult pathologies to diagnose and treat [3,4]. Its etiologies include traumatic nerve injuries, inflammatory and metabolic disorders, and channelopathies such as sodium channel mutations [5,6].

Depending on lesion location, neuropathic pain is classified as peripheral or central, occurring in conditions such as trigeminal neuralgia, painful polyneuropathy, radiculopathy, postherpetic neuralgia [7], spinal cord injury, stroke, and multiple sclerosis [8].

Despite extensive research, the pathophysiological mechanisms of neuropathic pain remain only partially understood. Globally, 20–30% of the population develops chronic pain [2,9]. Peripheral neuropathic pain represents the most cases and arises from disorders such as diabetic neuropathy, nerve compression syndromes, chemotherapy-induced neuropathy, and post-surgical neuropathic pain [1]. In contrast, central neuropathic pain is less common but often associated with great clinical severity and resistance to treatment, particularly in conditions such as stroke (7–10%), spinal cord injury (up to 40–70%), and multiple sclerosis (~30%) [10].

One of the main limitations in neuropathic pain management is the lack of objective assessment tools; thus, diagnosis still relies primarily on patient-reported self-assessment scales [10,11]. This frequently results in diagnostic delays and misdiagnoses, negatively affecting patients’ quality of life, treatment efficacy and socioeconomic costs [12,13,14].

Therefore, increasing attention has been directed toward the identification of biological markers that could provide objective indicators for pain assessment and improve the understanding of pain-related mechanisms [15,16].

Although several candidate biomarkers have been proposed, none has yet achieved sufficient clinical validation for routine use [17]. Proposed biomarkers include inflammatory mediators, such as IL-6, TNF-α, and IL-1β; neurotrophic factors, including brain-derived neurotrophic factor (BDNF); and markers of neuronal injury, such as neurofilament light chain (NfL) [18]. Neuroimaging techniques, including magnetic resonance imaging (MRI) and positron emission tomography (PET), as well as electroencephalography (EEG) and magnetoencephalography (MEG), have also provided insights into structural and functional alterations associated with pain processing.

More recently, omics-based approaches, including proteomics and metabolomics [19], together with immune-related markers such as alterations in lymphocyte subsets and receptor expression profiles, have emerged as potential indicators of neuroimmune involvement in pain states [20,21,22].

Nevertheless, their clinical applicability remains limited due to insufficient sensitivity, specificity, reproducibility, and the marked heterogeneity of neuropathic pain mechanisms [20].

In recent years, our research group has focused on the expression of the µ-opioid receptor (MOR) in immune cells as a potential objective biomarker for chronic pain, especially in B lymphocytes and natural killer (NK) cells. We previously demonstrated that the percentage of MOR-expressing B cells (MOR + B cells %) is significantly reduced in patients with fibromyalgia and osteoarthritis experiencing moderate-to-severe pain compared with pain-free individuals. Similarly, patients with fibromyalgia exhibit a lower percentage of MOR-positive NK cells, suggesting a common mechanism that may impair peripheral opioid-mediated pain control [20,21,22]. These findings suggest the hypothesis that MOR expression in immune cells could represent an innovative, measurable and pragmatic tool for chronic pain management. Its clinical integration would be supported by an easily accessible peripheral blood draw, short processing times, and reliable stability, ultimately allowing for objective patient evaluation, risk stratification, and long-term follow-up.

It is well known that the opioid receptors are primarily G protein-coupled receptors that signal through Gi/o proteins. Upon activation, MOR signaling leads to inhibition of adenylate cyclase activity and a consequent reduction in intracellular cyclic AMP (cAMP) levels, resulting in the modulation of protein kinase A (PKA)-dependent pathways. These intracellular changes can influence downstream signaling cascades, including mitogen-activated protein kinase (MAPK) pathways, as well as calcium-dependent processes that regulate NK cell degranulation and cytokine production. Collectively, these pathways may contribute to alterations in NK cell cytotoxic function and immune–neural communication in the context of neuropathic pain [11,23]. Since NK cell function depends on the balance between activating and inhibitory signals, MOR-mediated modulation could have important functional consequences.

NK cells are large granular lymphocytes of the innate immune system, playing a crucial role in the control of infections, cancer, and transplant rejection [24,25,26].

They eliminate virus-infected or transformed cells and secrete granzymes and cytokines, such as IFN-γ, which modulate other immune responses [27,28].

NK cell activity is regulated by a complex balance of activating and inhibitory signals mediated by germline-encoded receptors that recognize ligands expressed on both normal and transformed cells [29]. Their activation is tightly controlled by inhibitory receptors specific for the Major histocompatibility complex (MHC) class I molecules, including killer cell immunoglobulin-like receptors (KIRs) in humans, Ly49 family receptors in mice, and NKG2A/CD94 receptors shared by both species [30].

Through these inhibitory mechanisms, NK cells are able to detect the “missing self” phenomenon, characterized by reduced or absent expression of self MHC class I molecules on potential target cells [31,32]. In mice, the Immunoreceptor Tyrosine-based Inhibitory Motif (ITIM), located within the cytoplasmic domain of Ly49 receptors, such as Ly49A, becomes phosphorylated upon engagement with MHC class I molecules, including H-2D^d^, a murine MHC class I molecule, thereby mediating inhibitory signaling. This event recruits the phosphatase Shp-1, which plays a crucial role in inhibiting activating signaling in NK cells [33,34,35]. Once recruited, Shp-1 dephosphorylates key proteins involved in signal transduction, preventing the activation of intracellular pathways responsible for cytokine secretion and cytotoxic granule release. In this way, Shp-1 enables NK cells to distinguish healthy cells from infected or transformed cells, ensuring that activation occurs only in the presence of “missing self” signals [36].

Increasing evidence indicates that NK cells contribute to the neuroimmune mechanisms underlying peripheral neuropathic pain. Following peripheral nerve injury, immune activation leads to the recruitment and modulation of NK cells, which can interact with neurons and other immune populations. In addition to indirect pro-inflammatory effects, NK cells may exert direct cytotoxic actions on sensory neurons through receptor–ligand interactions, including NKG2D-mediated recognition of stress-induced ligands expressed by injured neurons. These mechanisms can contribute to peripheral sensitization and to the development or maintenance of neuropathic pain. However, the role of NK cells appears to be complex and context-dependent, as both pathogenic and protective functions have been described [37].

Moreover, opioids are known to affect immune cell activity by exerting immunosuppressive effects, including the down-regulation of NK cell activity [11,31]. Notably, patients with neuropathic pain exhibit a reduction in NK cell function [38], further supporting the investigation of opioid receptor expression in these cells and its potential role as an objective indicator for pain monitoring.

Based on these observations, in the present study, we investigated the effects of peripheral nerve injury induced by unilateral chronic constriction injury (CCI) of the sciatic nerve [39], on MOR expression and localization in NK cells in a murine model of neuropathic pain. In addition, we evaluated NK cell cytotoxic function by analyzing degranulation (CD107a, also known as lysosomal-associated membrane protein 1, LAMP-1), GRZ-A release, and IFN-γ production as indicators of immunomodulatory function [40,41].

## 2. Results

### 2.1. Development of Hyperalgesia and Allodynia Following CCI of the Sciatic Nerve

Neuropathic pain causes debilitating symptoms, such as spontaneous pain, allodynia, hyperalgesia, and dysesthesia/paresthesia [42,43].

**Figure 1:**
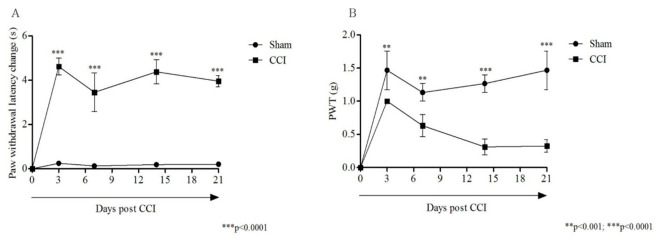
Graphs showing the neuropathic pain effect in C57BL/6j mice on paw withdrawal latency after thermal stimulation (**A**) and PWT after mechanical stimulation (**B**), assessed over a 21-day period. Each point represents the mean withdrawal latency (s) (**A**) or withdrawal threshold (PWT) (g) (**B**). Results are expressed as mean ± SEM for 3 different experiments (**A**). *** *p* < 0.0001 vs. sham; (**B**) ** *p* < 0.001 vs. CCI; *** *p* < 0.0001 vs. CCI.

In this study, we observed that CCI significantly reduced both thermal withdrawal latencies and mechanical withdrawal thresholds (PWT) in the ipsilateral hind paw, measured by the Hargreaves method (Figure 1A) and Von Frey test (Figure 1B), respectively, when compared to sham-treated mice. No changes were observed in the contralateral paw withdrawal.

Although stress-related effects associated with surgery cannot be completely excluded, sham and CCI mice underwent identical surgical procedures, anesthesia, handling, and experimental manipulation, differing only in the presence of sciatic nerve ligation. Therefore, potential confounding effects related to procedural stress were equally distributed between groups.

### 2.2. Decreased NK Cell Percentage and Surface MOR Expression Following CCI

**Figure 2:**
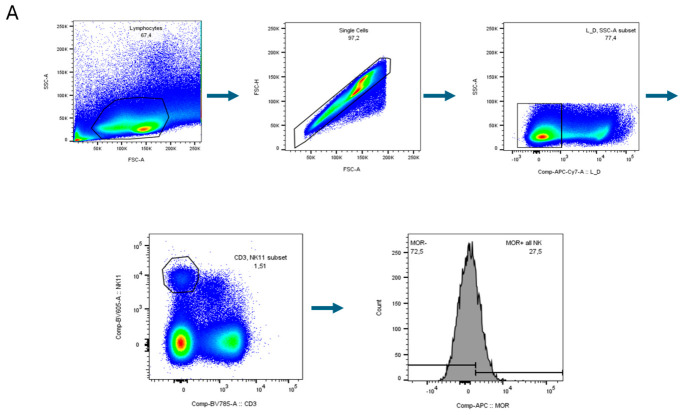

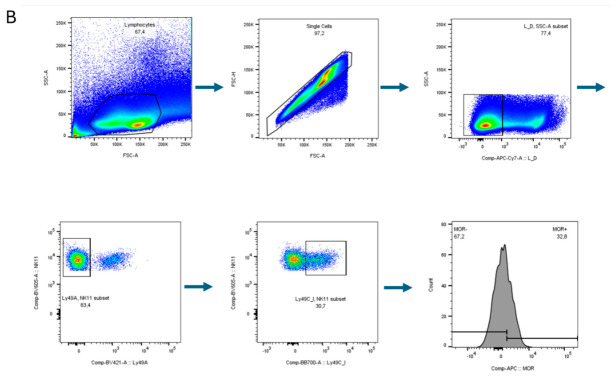
(**A**): Flow cytometry analysis for measuring expression of the MOR of B6 mice. Lymphocytes were incubated with cell-surface receptor staining. MOR cell-surface expression on total NK cells. The gating strategy was performed as follows: lymphocytes were first identified based on SSC-A versus FSC-A parameters; singlets were then selected using FSC-H versus FSC-A discrimination; viable cells were identified by Live/Dead staining using SSC-A versus APC-Cy7 Live/Dead signal. NK cells were subsequently identified as NK1.1^+^CD3^−^ cells and analyzed for MOR cell-surface expression. One representative experiment. (**B**): Flow cytometry analysis for measuring expression of the MOR of B6 mice. Lymphocytes were incubated with cell-surface receptor staining. Events were gated on live singlet lymphocytes, CD3^−^NK1.1^+^ NK cells, and further gated to exclude cells expressing Ly49A, an inhibitory receptor that recognizes H-2D^d^. The gating strategy was performed as follows: lymphocytes were first identified based on SSC-A versus FSC-A parameters; singlets were then selected using FSC-H versus FSC-A discrimination; viable cells were identified by Live/Dead staining using SSC versus APC-Cy7 Live/Dead signal. NK cells were subsequently identified as NK1.1^+^CD3^−^ cells, and further gated to exclude cells expressing Ly49A, an inhibitory receptor that recognizes H-2D^d^, and Ly49C/I inhibitory receptors and then analyzed for MOR cell-surface expression. One representative experiment.

**Figure 3:**
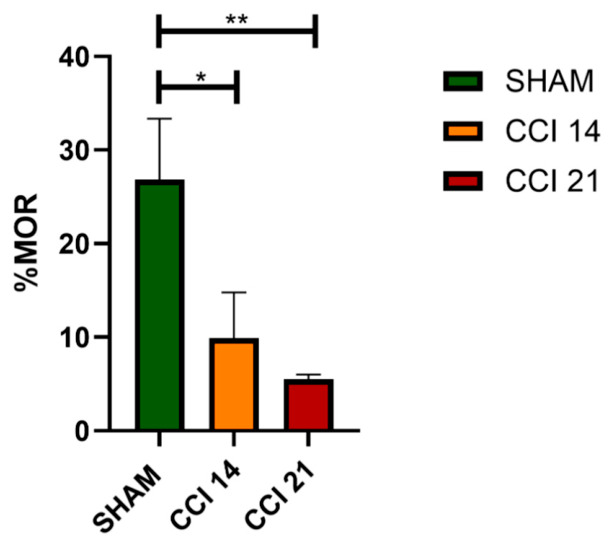
Flow cytometric analysis to measure MOR expression on total NK cells from C57BL/6j mice spleen at 14 and 21 days after CCI. Results are expressed as mean ± SEM for 3 different experiments. Results are expressed as mean ± SEM for 3 different experiments. Events were gated on live singlet lymphocytes, CD3−NK1.1+. * *p* < 0.05, ** *p* < 0.005 vs. sham.

**Figure 4:**
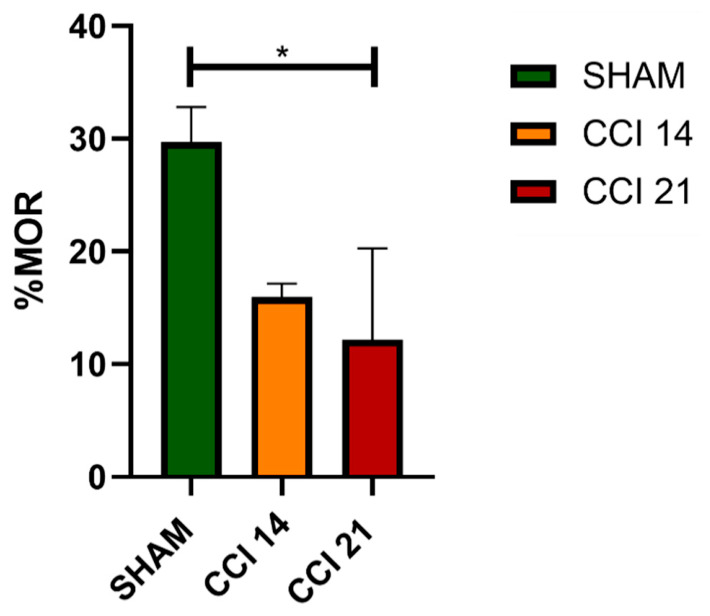
Flow cytometric analysis to measure MOR expression on Ly49C/I^+^ NK cells from C57BL/6j mice spleen at 14 and 21 days after CCI. Results are expressed as mean ± SEM for 3 different experiments. Events were gated on live singlet lymphocytes, CD3^−^NK1.1^+^, and further gated to exclude cells expressing Ly49A. * *p* < 0.05 vs. sham.

Spleen cells from mice were analyzed to determine the percentage of NK cells expressing MOR on their surface. Two gating strategies were used: one based on the total NK cell population (CD3-NK1.1+) and the other using the inhibitory marker Ly49C (Figure 2A and Figure 2B, respectively). Specifically, using the gating strategy for the total NK cell population isolated from the spleen, FACS analysis revealed a significant reduction in the percentage of MOR-expressing NK cells in CCI mice at 14 and 21 days after sciatic nerve ligation compared to sham controls (Figure 3). A similar result was observed by flow cytometric analysis of MOR expression in Ly49C/I^+^ NK cells isolated from the spleens of C57BL/6j mice at 14 and 21 days after CCI (Figure 4).

**Figure 5:**
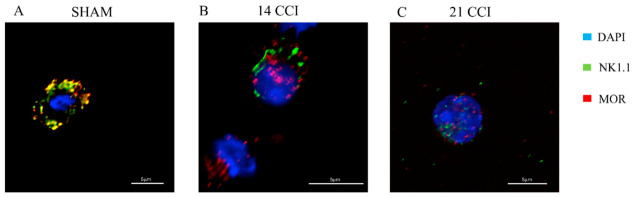
Confocal microscopy analysis of MOR localization in NK cells from B6 mice at ×60 magnification with different digital zooms. DAPI (blue) stains nuclei, NK1.1 (green) identifies NK cells, and MOR (red) indicates μ-opioid receptor expression. The yellow signal observed in merged images, particularly in panel A, results from the overlap of NK1.1 (green) and MOR (red) fluorescence, indicating MOR expression in NK1.1-positive NK cells. Sham mice (**A**), CCI group for 14 days (**B**), CCI for 21 days (**C**). (Scale bar = 5 μm.)

To evaluate the localization of the MOR in NK cells, we performed confocal microscopy analysis using splenic lymphocyte cells from sham mice and from mice 14 or 21 days after CCI. Our aim was to investigate whether it could be internalized into the cytoplasm, potentially explaining the quantitative reduction observed in CCI mice. Interestingly, we observed MOR expression predominantly on the cell surface in splenic lymphocyte cells from sham mice (Figure 5A). In contrast, in splenic lymphocyte cells from mice subjected to CCI for 14 or 21 days, MOR was mainly localized intracellularly (Figure 5B,C). This finding supports the FACS analysis results, which showed a decreased percentage of NK cells expressing membrane-bound MOR following CCI. The full panel is shown in Supplementary Figure S1.

### 2.3. Correlations Between Mu-Positive NK Cells and Pain-Related Parameters

Table 1 reports the correlation coefficients between the percentage of Mu+ NK cells and pain-related parameters, including allodynia and hyperalgesia in mice. Pearson’s correlation analysis revealed a strong and statistically significant negative correlation between MOR expression and plantar test results (r = −0.932; *p* < 0.001; *n*= 12) (Table 1). This finding indicates that higher expression of the μ-opioid receptor (MOR) is associated with a reduced nociceptive response (i.e., increased pain sensitivity), whereas lower MOR expression is associated with an increased pain response in the plantar test. Moreover, a strong and significant positive correlation was observed between MOR expression and Von Frey test responses (r = 0.735; *p* = 0.006; *n*= 12) (Table 1). These results indicate that higher MOR expression is associated with increased mechanical withdrawal thresholds (i.e., reduced mechanical sensitivity), whereas lower MOR expression corresponds to enhanced mechanical hypersensitivity in the Von Frey test.

### 2.4. CCI Induces a Significant Decrease in Unstimulated CD107a Expression in NK Cells Compared to Sham Controls

**Figure 6:**
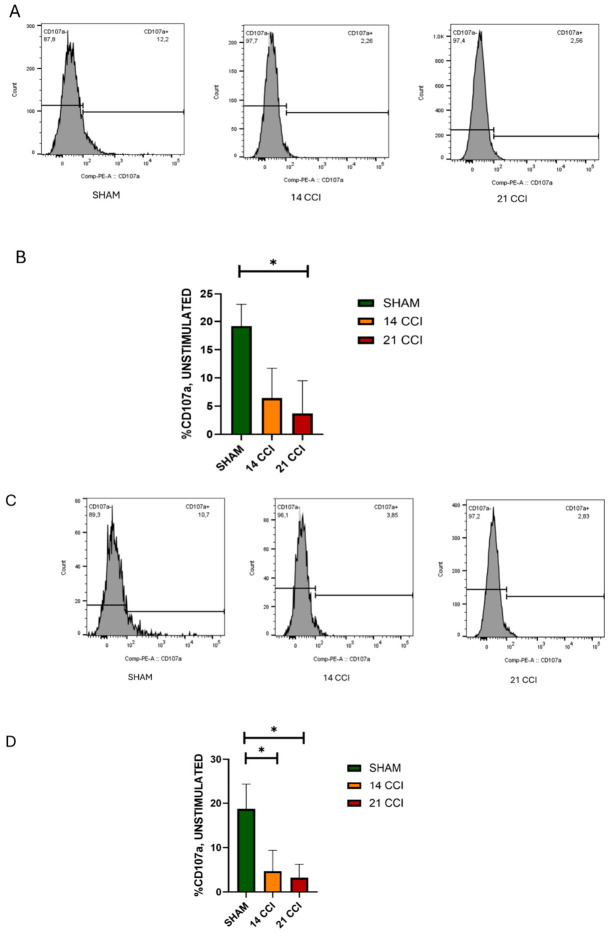
(**A**): Flow cytometry analysis for measuring granular release in subsets of B6. The splenic lymphocytes were incubated for 3 h at 37 °C in the presence of CD107a Ab, followed by cell-surface receptor staining 14 and 21 days after CCI. Results are expressed as mean ± SEM, for 6 mice per group. Events were gated on live singlet lymphocytes, CD3^−^NK1.1^+^ NK cells (**A**,**B**), under unstimulated conditions. * *p* value < 0.05. One representative example of gating, showing expression of CD107a Ab. (**B**): Events were gated on live singlet lymphocytes, CD3−NK1.1+ NK cells, and further gated to exclude cells expressing Ly49A. CD107a cell-surface expression on Ly49C/I subpopulation at 14 and 21 days after CCI (**C**,**D**), under unstimulated conditions. Results are expressed as mean ± SEM, for 6 mice per group. * *p* value< 0.05. One representative example of gating, showing expression of CD107a Ab (**C**).

LAMP-1, also known as CD107a, is a highly glycosylated transmembrane protein primarily located in lysosomes. In NK cells and cytotoxic T lymphocytes (CTLs), CD107a is abundantly present within lytic granules. Here, we observed that CCI after 14 or 21 days causes a significant reduction in CD107a production compared with sham, under unstimulated conditions, from total NK cells (CD3-NK1.1^+^; Figure 6A) and in Ly49C NK cells (Figure 6B).

As positive control, stimulation with PMA/ionomycin induced a marked increase in the percentage of CD107a surface expression from the total NK population in sham controls compared with CCI samples, with sham animals showing significantly higher CD107a expression than CCI mice, particularly at 21 days post-injury. By contrast, CD107a surface expression within the Ly49C/I-positive NK cell subpopulation also showed an increased percentage in sham controls compared with CCI samples, although the difference between sham and CCI groups did not reach statistical significance (compared to those unstimulated; Supplementary Figures S2 and S3).

### 2.5. Extracellular Release of GRZ-A from NK Cells After CCI

**Figure 7:**
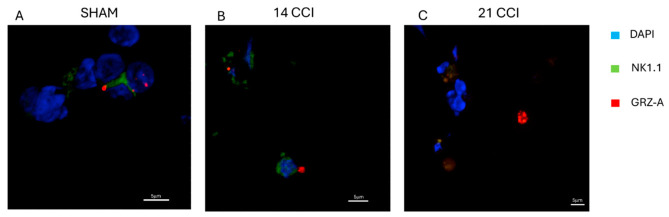
Confocal microscopy analysis of GRZ-A receptor localization and release in NK cells from B6 mice at ×60 magnification with different digital zooms. DAPI (blue) nucleus, NK.1.1 (green) to NK cells marker; GRZ-A (red) indicating granule localization, sham mice (**A**), CCI group for 14 days (**B**), CCI for 21 days (**C**). (Scale bar = 5 μm).

Confocal microscopy analysis was performed on splenic lymphocytes isolated from sham mice and from mice at 14 and 21 days post-CCI to examine GRZ-A localization within NK cells. In splenic lymphocytes from sham mice, GRZ-A was predominantly localized intracellularly. In contrast, in splenic lymphocytes from mice 14 or 21 days after CCI, GRZ-A was mainly observed in the extracellular space, suggesting its release from NK cells in response to injury and inflammation (Figure 7). The full panel is shown in Supplementary Figure S4.

### 2.6. Interferon-Gamma (IFNγ) Production Decreases in Total NK Cells and Ly49C/I Subsets Following CCI

IFNγ is primarily produced by activated T-helper (TH)1 cells and NK cells, with smaller contributions from macrophages, dendritic cells (DCs), and B cells. In NK cells, IFNγ production is triggered by type I interferons (IFNα and IFNβ) via signal transducer and activator of transcription 4 (STAT4)-dependent signaling pathways [34,35].

We investigated the percentage of IFNγ-producing NK cells in sham mice and in mice at 14 or 21 days after CCI, considering both total NK cells and the Ly49C subtype. Under resting conditions, IFNγ production in sham mice was negligible in both NK cells and the Ly49C subtype, with values close to zero. A modest increase in IFNγ expression was observed in sham mice compared with CCI mice at 14 and 21 days; however, this increase did not reach statistical significance (Supplementary Figure S5A; NK cells). In contrast, within the Ly49C/I^+^ subset, a significant increase was observed.

**Figure 8:**
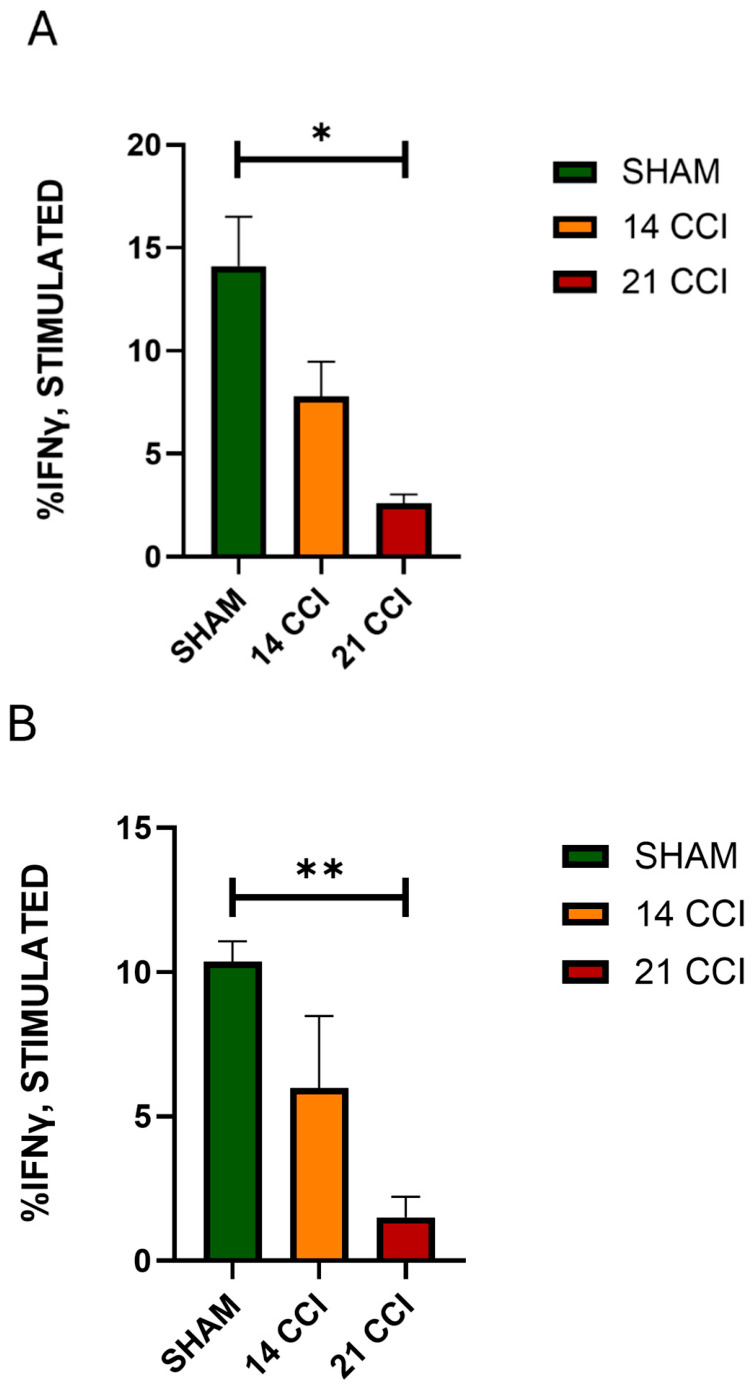
(**A**): Flow cytometry analysis for measuring production of IFNγ, in stimulated conditions with PMA/I. Events were gated on live singlet lymphocytes, CD3−NK1.1+ NK cells at 14 and 21 days after CCI. Stimulated conditions with PMA/I for 3 h. Results are expressed as mean ± SEM, for 6 mice per group. * *p* value < 0.0050. (**B**): Flow cytometry analysis for measuring cytokine release in B6 mice at 14 and 21 days after CCI. The splenic lymphocytes were incubated with the full panel of antibodies for 2–3 h at 37 °C. We performed surface staining followed by intracellular staining in the presence of IFNγ Ab. Events were gated on live singlet lymphocytes, CD3−NK1.1+ NK cells, and further gated to exclude cells expressing Ly49A. Intracellular expression of IFNγ on the Ly49C/I gate. Stimulated conditions with PMA/I for 3 h. Results are expressed as mean ± SEM, for 6 mice per group. ** *p* value < 0.0050.

Following stimulation with PMA/I, NK cells from sham mice exhibited a robust IFNγ response, both in total NK cells and the Ly49C subtype in the sham group (Figure 8A and Figure 8B, respectively). In contrast, NK cells from CCI mice at 14 and 21 days did not show a substantial increase compared to sham, suggesting a blunted or plateaued activation response in this model (Figure 8A and Figure 8B, respectively).

## 3. Discussion

Neuropathic pain is a complex and disabling condition with high prevalence and substantial socioeconomic impact [44,45].

Peripheral neuropathy is a common neurological disorder worldwide, affecting approximately 1–3% of the general population and up to 7–8% of older adults. Its prevalence is particularly high among individuals with diabetes, one of the leading causes of peripheral neuropathy. Western populations show different values reported in several European countries, including approximately 10% in France and Italy, 11% in Germany, 13% in Portugal, and up to 23% in Spain [44,46]. In the United States, population-based studies have estimated a prevalence of approximately 13–14% among adults aged 40 years and older, increasing to nearly 28% in individuals with diabetes. Similarly, data from Japan indicate that diabetic symmetric sensorimotor polyneuropathy affects approximately one-third of patients with type 2 diabetes. Overall, the prevalence of diabetic peripheral neuropathy varies considerably across populations and diagnostic criteria, ranging from less than 10% to over 40% in clinical and epidemiological studies [2,47,48].

To our knowledge, our group was the first to propose the modulation of the MOR in immune cells as a potential peripheral biomarker of chronic pain. Accordingly, we investigated the association between MOR expression in peripheral immune cells and different chronic pain conditions in patient populations. This aspect, which represents a key innovative element of the study, has now been more explicitly highlighted throughout the revised manuscript.

Overall, neuropathic pain is associated with a substantial clinical and economic burden due to its chronicity, diagnostic complexity, and limited response to available treatments. In the United States, the annual cost of chronic pain exceeds 600 billion dollars, with neuropathic pain representing a major contributor to this socioeconomic burden [49,50,51].

At present, peripheral neuropathic pain is recognized as a neuroimmune disorder in which immune, glial, and neuronal cells interact to sustain peripheral and central sensitization processes [52].

In this context, NK cells represent relevant mediators of neuroinflammatory signaling through cytokine release and cytotoxic effector functions, influencing neuronal excitability and glial activation, and thereby contributing to the initiation and maintenance of neuropathic pain [52,53,54,55].

Under physiological conditions, NK cells participate in the activation and apoptosis of inflammatory cells such as neutrophils and eosinophils. Abnormalities in NK cell function include a decreased ability to degranulate, a limited ability to synthesize and secrete IFNγ and TNFα, and a reduced expression of CD314 (NKG2D), CD335 (NKp46) and CD337 (NKp30) receptors [54,55,56,57].

Evidence also implicates the opioid system in neuroimmune regulation [11,21].

NK cells expressing MOR play a central role in innate immunity, mediating cytotoxic activity against infected or transformed cells and regulating adaptive immune responses.

In particular, expression of MOR in NK cells regulates cytotoxic activity, cytokine production, and neuroimmune interactions [11].

Accordingly, alterations in MOR expression or signaling could therefore contribute to dysfunctional neuroimmune signaling associated with chronic pain maintenance [58].

Dysregulation of NK cell activity, including reduced cytotoxicity and altered expression of activating and inhibitory receptors, has been implicated in chronic pain states, potentially contributing to persistent inflammation and impaired immune surveillance [59,60].

Within this context, NK-derived cytokines and cytotoxic mediators can influence neuronal and glial activity. MOR signaling in NK cells may therefore directly modulate immune effector functions, and its dysregulation may contribute to dysfunctional neuroimmune communication in peripheral sensitization [58,61].

Pathways linking immune activation, inflammation and neuropathic pain are well established, particularly in relation to neuroimmune signaling and peripheral sensitization [62,63,64,65]. Although morphine is considered primarily an MOR agonist, it has broad pro-oxidant and immunomodulatory effects, which lead to hyperalgesic pain [11,66]. In experimental animal models, it has been shown to suppress immune responses, including NK cell cytotoxicity, lymphocyte proliferation, antibody production, and phagocytic activity. These effects involve both direct immune modulation and neuroendocrine mechanisms, including activation of the hypothalamic–pituitary–adrenal axis and glucocorticoid release, as well as IL-1β-dependent pathways [38,65,67,68,69].

However, the mechanisms linking MOR expression percentage in NK cells to the development and maintenance of chronic pain are still poorly understood.

To address this issue, we explored the involvement of NK cells and MOR in neuropathic pain using a murine model of unilateral chronic constriction injury (CCI) of the sciatic nerve over a 21-day period. This model induces both mechanical and thermal hypersensitivity, as confirmed by Von Frey and Hargreaves tests. CCI mice exhibited significant nociceptive hypersensitivity compared with sham controls.

Concomitantly, CCI induced a significant decrease in the percentage of NK cells expressing MOR at both 14 and 21 days after injury compared with sham animals. Interestingly, this reduction was accompanied by a marked change in receptor localization. While in sham conditions, MORs were mainly distributed on the cell surface; in neuropathic mice, it was predominantly found within the cytoplasmic compartment. Notably, MOR is known to undergo rapid endocytosis upon activation by endogenous or exogenous opioids, leading to its accumulation in intracellular compartments [20]. In the context of neuropathic pain, increased levels of endogenous opioids and sustained receptor engagement may promote this process, thereby reducing surface receptor availability [21].

Importantly, MOR expression was strongly correlated with behavioral outcomes, showing a negative correlation with plantar test responses and a positive correlation with Von Frey thresholds, suggesting that MOR expression contributes to the modulation of nociceptive sensitivity [54].

To further investigate NK cell regulation, we analyzed MOR expression within the inhibitory Ly49C/I NK cell subset, as well as functional parameters including degranulation and interferon-γ production.

In C57BL/6 mice, Ly49C and Ly49I receptors contribute to NK cell education through the recognition of functional competence and self-tolerance [70,71]. Alterations in the balance between activating and inhibitory receptors can lead to NK cell dysfunction, a phenomenon described in chronic infections and cancer [71].

Indeed, qualitative confocal analysis revealed extracellular GRZ-A release following CCI, indicating active spontaneous degranulation, as shown by the CD107a assay. NK cell cytotoxic activity is primarily mediated through the release of perforin and granzymes [72], as well as on death receptors on target cells and their corresponding ligands on NK cells, and antibody-dependent cell-mediated cytotoxicity (ADCC) [73,74].

In parallel, the surface expression of CD107a correlates with altered degranulation, confirming impaired NK cell function. In particular, we detected reduced CD107a expression in NK cells from CCI mice at both 14 and 21 days compared with sham controls under unstimulated conditions in total NK cells and specifically in the Ly49C/I^+^ NK cell subset. This reduction in degranulation reflects altered NK cell activation and supports their involvement in the immune response associated with neuropathic pain.

Multiple mechanisms contribute to this dysfunctional phenotype [75], including the dysregulation of NK cell receptor signaling, with a downregulation of activating receptors and upregulation of inhibitory receptors, altering receptor balance and disrupting the ability of NK cells to recognize and eliminate target cells effectively [29,54,75].

Furthermore, NK cells play a crucial role in protective IFN-γ production during infection and inflammation [76,77].

Interestingly, we observed a modulation of IFN-γ levels in NK cells 14 and 21 days after CCI. Specifically, following PMA/I stimulation, NK cells from CCI mice showed significantly reduced IFN-γ production compared with sham groups, indicating a state of NK cell hyporesponsiveness, characterized by reduced effector cytokine production. In states such as chronic infections or in many tumors, NK cells undergo progressive functional impairment characterized by reduced cytotoxicity, impaired cytokine production, and altered receptor expression profiles [78,79,80,81,82].

Collectively, these data support a model in which peripheral nerve injury is associated with reduced MOR availability on NK cells, receptor internalization, and impaired NK cell effector function. Rather than a direct causal mechanism, our data suggested a concurrent phenomenon between altered opioid signaling and NK cell dysfunction in neuropathic pain states [54].

Although NK cells are implicated in neuroimmune mechanisms involved in peripheral and central sensitization, supporting interest in immunomodulatory therapeutic strategies [54], clinical studies directly correlating NK cell populations, pain parameters, and opioid receptor expression in humans remain limited [11,15,17,20,21,22].

Clinically, chronic pain is associated with reduced function and increased reliance on analgesics, yet assessment remains largely subjective. This underscores the need for objective biomarkers to improve diagnostic accuracy, stratify patients, and guide therapeutic strategies.

It is well known that opioid receptors are expressed not only in neurons but also in immune cells, including NK cells, where their dysregulation may contribute to reduced analgesic responsiveness in chronic neuropathic pain states [20,83]. In this context, our research group has focused on MOR-expressing immune cells as potential candidate biomarkers of pain in clinical trials of patients with chronic pain, such as osteoarthritis and fibromyalgia [15]. We previously demonstrated an inverse relationship between MOR expression and pain severity in B and NK cell populations [11,15,17,20,22].

Pain-free individuals exhibited significantly higher percentages of MOR-positive (Mu+) B and NK cells compared with patients with chronic pain, and lower percentages correlate with higher pain severity [17,21]. More specifically, we showed a significant association between MOR expression in immune cells and clinical pain phenotypes, suggesting that these cells may act not only as effector immune cells but also as potential biomarkers of pain modulation [20].

Furthermore, these data appear stable over time, supporting the reliability of MOR as an indicator of widespread chronic pain in patients with fibromyalgia [15].

Notably, these clinical observations are supported by our preclinical findings in the well-established CCI mouse model, where chronic neuropathic pain was associated with significant alterations in immune cell function and MOR expression. The concordance between clinical and preclinical data strengthens the hypothesis that immune-cell MOR modulation represents a biologically relevant mechanism underlying chronic pain and supports its potential role as a peripheral biomarker.

If validated, MOR-expressing immune cell frequency could provide an objective and reproducible measure of chronic pain, particularly valuable in patients unable to reliably self-report symptoms and may also have applications in medico-legal evaluation. Specifically, such a biomarker could have significant applications in medico-legal contexts by supplying reliable scientific evidence to support the evaluation of chronic pain in legal controversies or disability assessments.

Importantly, the determination of this biomarker requires only a minimally invasive peripheral blood sample, making it easily accessible in routine clinical practice. Furthermore, its rapid turnaround time and measurement stability support its clinical applicability, representing a novel and practical approach for the objective evaluation, stratification, and longitudinal monitoring of patients with chronic pain. Findings from this mouse study provide additional support for our hypothesis and strengthen the concept of MOR expression on immune cells as a potential biomarker of pain, suggesting that neuroimmune modulation of opioid receptor signaling may contribute to the regulation of immune cell function in chronic pain states.

### Limitations of the Study

Some limitations of the present study should be considered.

All experiments were performed on splenic NK cells. Consequently, the observed alterations should be interpreted as indicators of systemic immunomodulation rather than direct evidence of local immune mechanisms occurring at the site of nerve injury or within the central nervous system.

In addition, only male mice were included in this study in order to reduce biological variability associated with hormonal fluctuations during the estrous cycle, which may influence immune responses, opioid receptor expression, and pain sensitivity. While the use of a single sex allowed for a more controlled evaluation of the investigated mechanisms, sex-dependent differences are increasingly recognized in both neuropathic pain and immune regulation, including NK cell function [74].

Finally, GRZ-A release was evaluated qualitatively by confocal microscopy without quantitative analysis of intracellular GRZ-A levels. Nevertheless, NK cell degranulation was independently assessed through CD107a surface expression, supporting the presence of functional alterations in NK cells.

## 4. Materials and Methods

### 4.1. Animals

Male C57BL/6j mice (Envigo, Indianapolis, IN, USA), eight weeks of age, weighing approximately 25 g, were used in this study, according to the Bennett model. All procedures involving mice were conducted in compliance with European Economic Community regulations (Directive 2010/63/EU; authorization number 32659.27), the NIH Guidelines for the Care and Use of Laboratory Animals, and Italian regulations on animal research protection (D.L. 26/2014), approved on 9 May 2023.

The minimum number of animals required to achieve statistical significance (*p* < 0.05), as established by the International Society for the Study of Pain guidelines, was used. All animals in each group were included in behavioral tests, flow cytometry, confocal microscopy, and degranulation and IFN-γ assays.

All animals were maintained under the same temperature conditions (21 °C ± 1 °C) and humidity (60% ± 5%), with food and water ad libitum and with 12 h light/dark cycles. To enhance animal welfare, each cage was provided with environmental enrichment. Experiments were performed between 7:00 a.m. and 10:00 a.m. in a quiet environment. For the entire experiment duration, the operator who executed the measurements was blinded to the different treatment groups. All substances, unless otherwise specified, were purchased from Sigma Aldrich (Milano, Italy) and dissolved in a physiological saline solution (0.9% sodium chloride).

### 4.2. Neuropathic Pain Induction

Under anesthesia induced by ketamine (75 mg/kg) and medetomidine (1 mg/kg), mice underwent surgery (sham or CCI). Anesthesia was administered during the perioperative phase (day 0) and equally across all experimental groups, after the baseline measurement, whereas our behavioral assessments were performed from day 3 post-surgery and after full recovery of the animals. Therefore, any transient pharmacological effects of anesthesia are expected to have been minimized and are unlikely to have affected the final experimental outcomes [84].

Briefly, after shaving and disinfecting the right thigh, the sciatic nerve was gently exposed through a small incision in the mid-lateral region.

Three loose ligatures, spaced 1 mm apart, were placed around the nerve using 6.0 silk sutures. Nociceptive responses were evaluated before surgery (on day 0) and on days 3, 7, 14, and 21 post-surgery. Mice were sacrificed on days 14 and 21 after CCI, and spleens were immediately collected and stored in phosphate-buffered saline (PBS) for subsequent analyses.

### 4.3. Behavioral Tests

Mechanical allodynia and thermal hyperalgesia were assessed at 0, 3, 7, 14, and 21 days after sciatic nerve ligation to evaluate nociceptive behavior. Hind paw sensitivity to mechanical stimuli was measured using calibrated Von Frey filaments (Ugo Basile, Varese, Italy).

Paw Withdrawal Threshold (PWT, g) values were determined using the Dixon up-and-down method [85], based on sequential increases and decreases in stimulus intensity (mechanical allodynia), whereas higher values reflect reduced sensitivity [86,87].

Before testing, mice were acclimatized for 15 min in Plexiglas cages equipped with a wire mesh floor prior to testing. Von Frey filaments were then applied perpendicularly to the medial plantar surface of the paw using increasing bending forces until the filament bent. Each filament was applied three times for 2 s per application.

An interval of 30 s was allowed between each application. A positive response was considered when mice exhibited paw withdrawal in at least three out of five filament applications, and the corresponding PWT was recorded. Mechanoallodynia was defined as a significant reduction (*p* < 0.05) in mechanical paw withdrawal threshold (g) compared with baseline forces (before treatment). Thermal hyperalgesia was assessed by the thermal radiation paw withdrawal test, as described by Hargreaves [88], using a maximum latency of 20 s to prevent tissue damage in unresponsive animals. Following a 15 min acclimatization period in a Plexiglas cage (Ugo-Basile, Varese, Italy), a high-intensity mobile projector bulb was used to apply thermal stimuli to the hind paws, alternating between left and right, from the lower chamber area. The withdrawal latency period of the paw was evaluated to the nearest tenth of a second with an electronic clock and thermocouple circuit. To prevent tissue damage, the test was stopped if no response occurred within 20 s. For each time point, data are presented as the delta change in withdrawal latency (s), obtained by subtracting the withdrawal latency of the right hind paw (ipsilateral, CCI-treated paw) from that of the left hind paw (contralateral paw). Results are expressed as changes in paw withdrawal latency.

### 4.4. Experimental Groups

Experimental groups were established as follows [89,90,91]:

Sham group: Animals (n = 6) underwent a surgical procedure to expose the sciatic nerve without any ligation.

CCI 14-day group: Animals (n = 6) underwent CCI to the sciatic nerve.

CCI 21-day group: Animals (n = 6) underwent CCI to the sciatic nerve.

### 4.5. Lymphocyte Isolation from Spleen

On 14 or 21 days after CCI, mice were sacrificed, and spleens were collected for the analysis of MOR percentage in NK cells. Splenocytes were selected in accordance with standard immunological protocols, as the spleen is the major secondary lymphoid organ and represents one of the most widely used sources for immune phenotyping and NK-cell characterization in murine studies, allowing for the recovery of sufficient amounts of immune cells for robust flow cytometric and immunological analyses. In contrast, peripheral blood collection in mice often yields a limited number of immune cells, which may not be sufficient for extensive multiparametric flow cytometry analyses. In addition, the spleen provides a reliable and well-characterized source of lymphocytes for flow cytometric analyses [92,93,94].

The mice spleens were crushed in Petri dishes using pestles in 3–4 mL of PBS with 1% Fetal bovine serum (FBS). Cells were resuspended in PBS with 1% FBS, then collected in 15 mL tubes and centrifuged at 1500 RPM for 5 min. After centrifugation, the supernatant was removed, and 1 mL of erythrocyte lysis buffer (ACK, containing NH4Cl, BioLegend, San Diego, CA, USA) was added to the pellet and subsequently incubated for 4 min at room temperature. After 4 min, 10 mL of PBS with 1% FBS was added, and the lysate was filtered through 40 μm nylon filters. After washing, cells were resuspended in approximately 10 mL of PBS, counted, and 2 × 10^6^ cells were used for analysis.

### 4.6. Degranulation Assay

NK degranulation was assessed with a flow cytometric assay measuring CD107a cell surface mobilization (release of lytic granules) after incubation for 3 h at 37 °C and 5% CO_2_. Spontaneous release (“leakage”) of lytic granules was measured without stimulation of cells, while Phorbol 12-myristate 13-acetate/Ionomycin (PMA/I) was added to positive controls. In short, cells were incubated in complete a-MEM medium supplemented with 5% fetal bovine serum (FBS), 100 IU/mL serum penicillin and 100 µg/mL streptomycin (100 μg/mL), Monensin (10 μg/mL, BioLegend), Brefeldin A (10 μg/mL Biolegend) and CD107a-PE mAb (Becton Dickinson, Franklin Lakes, NJ, USA). For positive controls, cells were stimulated with either a cell activation cocktail (BioLegends), containing (PMA/I) or 50 ng/mL PMA and 1 μg/mL Ionomycin. Cell acquisition was performed using an LSR Fortessa X20 flow cytometer (Becton Dickinson). Data analyses were done using Flowjo software 10.8.

### 4.7. Flow Cytometry Analysis

The cells obtained by lymphocyte isolation were incubated with different fluorochrome-conjugated Abs, specific for NK cells, in combination with the MOR antibody. All surface and intracellular staining steps were performed at 4 °C and in PBS complemented with 1% FBS. Fc Receptors (FcRs) were blocked by incubating cells with 0.5 mg/mL purified anti-mouse CD16/32 Antibody (BioLegend), and were later added. Samples were stained with NK1.1-BV605, CD3-BV786, Ly49A-BV421, Ly49C/I-BB700 (all Becton Dickinson) and MOR-APC (LSBio, Newark, CA, USA) antibodies for 20 min at 4 °C and centrifuged at 1500 RPM for 5 min. Following incubation, titration, and dilution in PBS at 4 °C for 30 min and subsequent washing, cells were processed for intracellular staining. For interferon-γ (IFN-γ-PE, Becton Dickinson), fixation and permeabilization were performed using the BD Cytofix/Cytoperm Plus Fixation/Permeabilization Kit (Becton Dickinson) followed by antibody labeling. Cells were fixed in 4.2% Paraformaldehyde (PFA) and analyzed the next day. Cells were acquired using an LSR Fortessa X20 flow cytometer (Becton Dickinson). Data analyses were done using FlowJo software 10.8. Unstained cells were used to discriminate doublets, and a sequential gating strategy was applied to exclude debris and dead cells, followed by the identification of lymphocytes based on forward and side scatter properties and LIVE/DEAD Aqua (1:400, Invitrogen, Carlsbad, CA, USA) to discriminate alive from dead cells. Fluorescence minus one (FMO) was used to control interactions between antibody conjugates; FMO (MOR^−^), FMO (IFN-γ^−^) and FMO (CD107a^−^) were used to set gates.

### 4.8. Immunofluorescence and Confocal Microscopy Analysis

Coverslips were treated with poly-L-lysine (10 mg/mL) and cross-linked using UV light for 50 min. Lymphocytes were then seeded onto the coverslips and cultured for 24 h. Subsequently, cells were fixed for 10 min at 37 °C with a Fixation and Permeabilization solution containing 4.2% formaldehyde (Becton Dickinson) and washed three times with Perm/Wash buffer (Becton Dickinson).

FcRs were blocked with purified anti-mouse CD16/32 antibody (1:100, BioLegend). Antibodies were diluted in Perm/Wash buffer for 60 min at 37 °C. Cells were incubated for 60 min at 37 °C with the following primary antibodies for MOR detection: anti-MOR (1:100, LSBio) and NK 1.1-BV605 (1:100, Becton Dickinson), followed by fluorescently labeled secondary antibodies, goat anti-rabbit IgG-Alexa Fluor 488 (1:500 Molecular Probes, Millipore, Burlington, MA, USA). To identify Granzyme-A, cells were incubated with (GRZ-A)-PE (1:100, Becton Dickinson) and NK 1.1-BV605 (1:100, Becton Dickinson). NK1.1 and GRZ-A were directly detected using fluorophore-conjugated primary antibodies. In contrast, for MOR-APC, a primary antibody was used, followed by a fluorophore-conjugated anti-rabbit secondary antibody, due to the low detectability of the primary antibody signal under confocal microscopy [22]. After staining, nuclei were labeled with DAPI (1:1000, Sigma, Kanagawa, Japan) in Perm/Wash buffer for 5 min at RT. Coverslips were mounted with FluoromountTM Aqueous Mounting Medium (Sigma F4680, Sigma-Aldrich, Burlington, MA, USA), and imaging was performed using a Nikon Eclipse Ti2 confocal microscope (Nikon, Tokyo, Japan) equipped with a VideoConfocal (ViCo) system. Secondary antibody combinations were chosen based on the primary antibodies employed.

### 4.9. Statistical Analysis

Values are presented as mean ± standard error of the mean (SEM), and the Kolmogorov–Smirnov test was applied to verify data normality.

After confirmation of normal distribution, differences between groups were analyzed using analysis of variance (ANOVA). For behavioral data (withdrawal latency and withdrawal threshold) obtained across multiple time points, two-way repeated measures ANOVA was used, followed by Bonferroni’s post hoc test for multiple comparisons. For flow cytometry and immunofluorescence data, comparisons among experimental groups (sham, CCI 14 days, CCI 21 days) were carried out using one-way ANOVA, followed by the Newman–Keuls post hoc test. All statistical analyses were performed in GraphPad Prism version 11.00 (GraphPad Software, Inc., San Diego, CA, USA), and statistical significance was accepted at *p*-value < 0.05.

## 5. Conclusions

In conclusion, in line with previous evidence, we observed reduced MOR expression in NK cells in CCI mice compared with sham controls, accompanied by a marked redistribution of the receptor from the cell surface to intracellular compartments. Functionally, NK cells from CCI mice exhibited impaired effector activity. We detected extracellular release of GRZ-A, indicating spontaneous degranulation, and surface expression of CD107a was reduced at both 14 and 21 days in total NK cells and specifically in the Ly49C^+^ subset, confirming altered degranulation capacity. Furthermore, IFN-γ production following stimulation was significantly lower in NK cells from CCI mice compared with sham controls, suggesting a state of NK cell functional impairment. Together, these findings indicate that neuropathic pain induces both MOR internalization and functional impairment in NK cells, linking the modulation of MOR expression percentage to decreased cytotoxicity and cytokine production.

## Figures and Tables

**Table 1:** Pearson’s correlation analysis between MOR expression and plantar test results (thermal hyperalgesia) and Von Frey filaments (mechanical allodynia). *n*= 12 animals (sham + CCI), respectively.

% MOR + NK Cells		
	Pearson’s Correlation	Significance (Two-Tailed)
Plantar test (thermal hyperalgesia)	−0.932 **	0.000
Von Frey filaments (mechanical allodynia)	0.735 **	0.006

** The correlation is significant at the 0.01 level (two-tailed).

## Data Availability

The original contributions presented in this study are included in the article/Supplementary Material. Further inquiries can be directed to the corresponding author.

## Supplementary Materials

The following supporting information can be downloaded at: https://www.mdpi.com/article/10.3390/ph19060933/s1, Figure S1: Events were gated on live singlet lymphocytes, CD3−NK1.1+ NK cells and in the presence of CD107a; PMA/Ionomycin was added to positive controls, followed by cell surface receptor staining 14 and 21 days after CCI. Events were gated on live singlet lymphocytes, CD3−NK1.1+ NK cells (A and B), under unstimulated conditions. Figure S2: Events were gated on live singlet lymphocytes, CD3−NK1.1+ NK cells, and further gated to exclude cells expressing Ly49A. CD107 cell surface expression on Ly49C/I at 14 and 21 days after CCI (C and D); PMA/Ionomycin was added to positive controls. Figure S3: Confocal microscopy analysis of the granule pattern in NK cells from sham B6 mice at ×60 magnification: SHAM, MARGE (Figure 1), DAPI (blue) nucleus (Figure 2), NK.1.1 (green) to NK cells marker (Figure 3); MOR (red) indicating receptor localization (Figure 4), for sham mice (A), CCI group for 14 days (B), CCI for 21 days (C). Figure S4: Confocal microscopy analysis of the granule pattern in NK cells from sham B6 mice, CTRL, MARGE (Figure 1). DAPI (blue) nucleus (Figure 2), NK.1.1 (green) to NK cells marker (Figure 3); GRZ-A (red) indicating granules localization (Figure 4), for sham mice (A), CCI group for 14 days (B), CCI for 21 days (C). Figure S5: (A) Flow cytometry analysis for measuring production of IFNγ, under stimulated conditions with PMA//I. (B) Flow cytometry analysis for measuring cytokine release in B6 mice at 14 and 21 days after CCI.

## Abbreviations

ACK	Ammonium-Chloride-Potassium (lysis buffer)
ADCC	Antibody-dependent cell-mediated cytotoxicity
ANOVA	Analysis of variance
Ab	Antibody
CCI	Chronic constriction injury
DAPI	4′,6-diamidino-2-phenylindole
DL	Decreto Legislativo
FACS	Fluorescence-activated cell sorting
FBS	Fetal bovine serum
FMO	Fluorescence minus one
GRZ-A	Granzyme A
IFN-γ	Interferon-gamma
ITIM	Immunoreceptor tyrosine-based inhibitory motif
KIR	Killer cell immunoglobulin-like receptor
LAMP-1	Lysosomal-associated membrane protein 1
MHC	Major histocompatibility complex
MOR	μ-Opioid receptor
NK cells	Natural killer cells
PBS	Phosphate-buffered saline
PFA	Paraformaldehyde
PMA/I	Phorbol 12-myristate 13-acetate/Ionomycin
PWT	Paw withdrawal threshold
RPM	Revolutions per minute
SEM	Standard error of the mean
STAT4	Signal transducer and activator of transcription 4
