# Supplementary material for: Modulation of Mu-Opioid Receptor Expression and Functional Impairment of Natural Killer Cells in Neuropathic Pain: Implications for Biomarker Discovery and Personalized Therapies
Source: Pharmaceuticals (Basel). 2026 Jun 13;19(6):933. doi: 10.3390/ph19060933 (PMC13306064; doi:10.3390/ph19060933)
Supplement: Supplementary file 1 [file pharmaceuticals-19-00933-s001.zip › pharmaceuticals-4288713-supplementary.pdf]

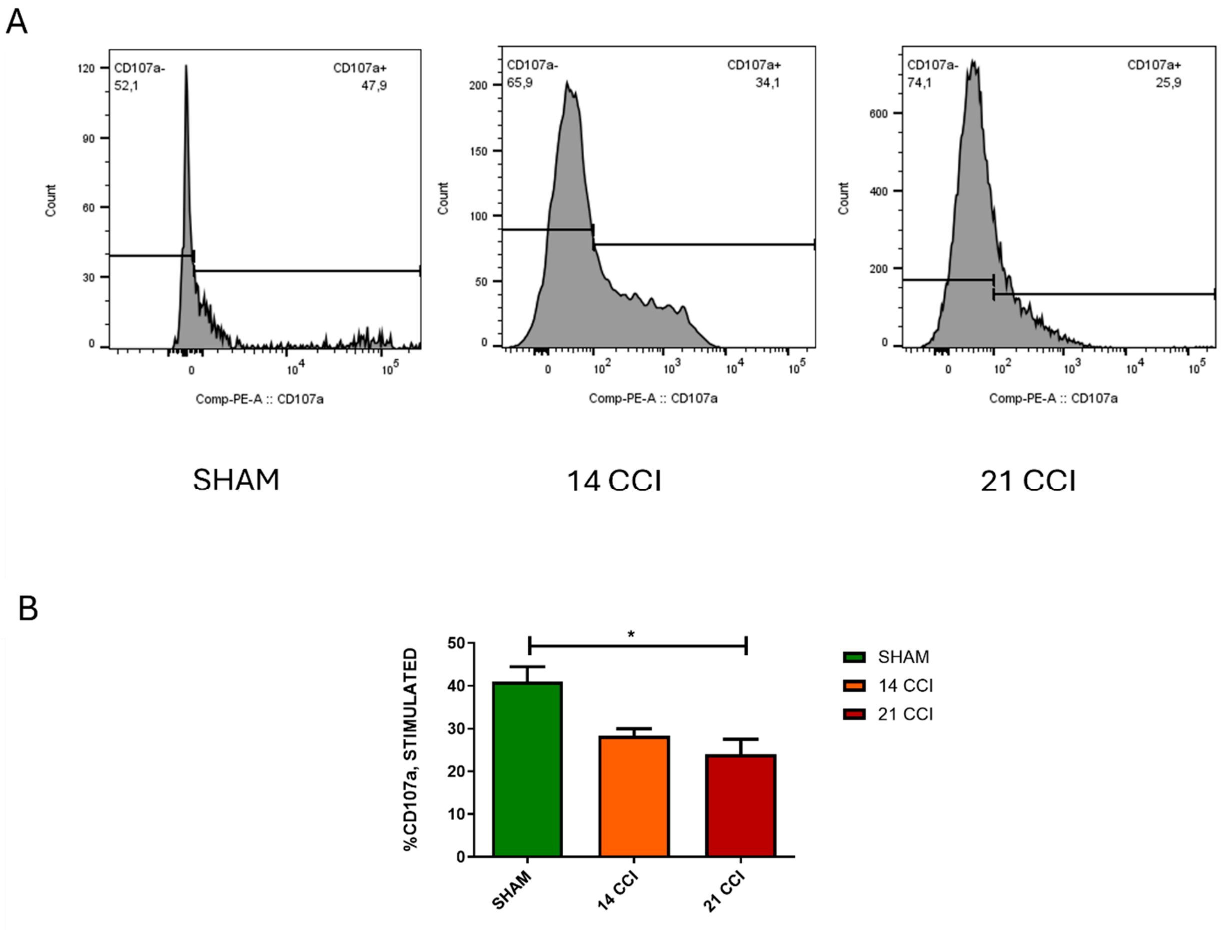

Figure S1

*Events were gated on live singlet lymphocytes, $CD3^{-}NK1.1^{+}$ NK cells and in the presence of CD107a; PMA/Ionomycin was added to positive controls, followed by cell surface receptor staining 14 and 21 days after CCI. Events were gated on live singlet lymphocytes, CD3−NK1.1+ NK cells (A and B), under unstimulated conditions. Results are expressed as mean ± SEM for 3 different experiments. *p value<0.050*

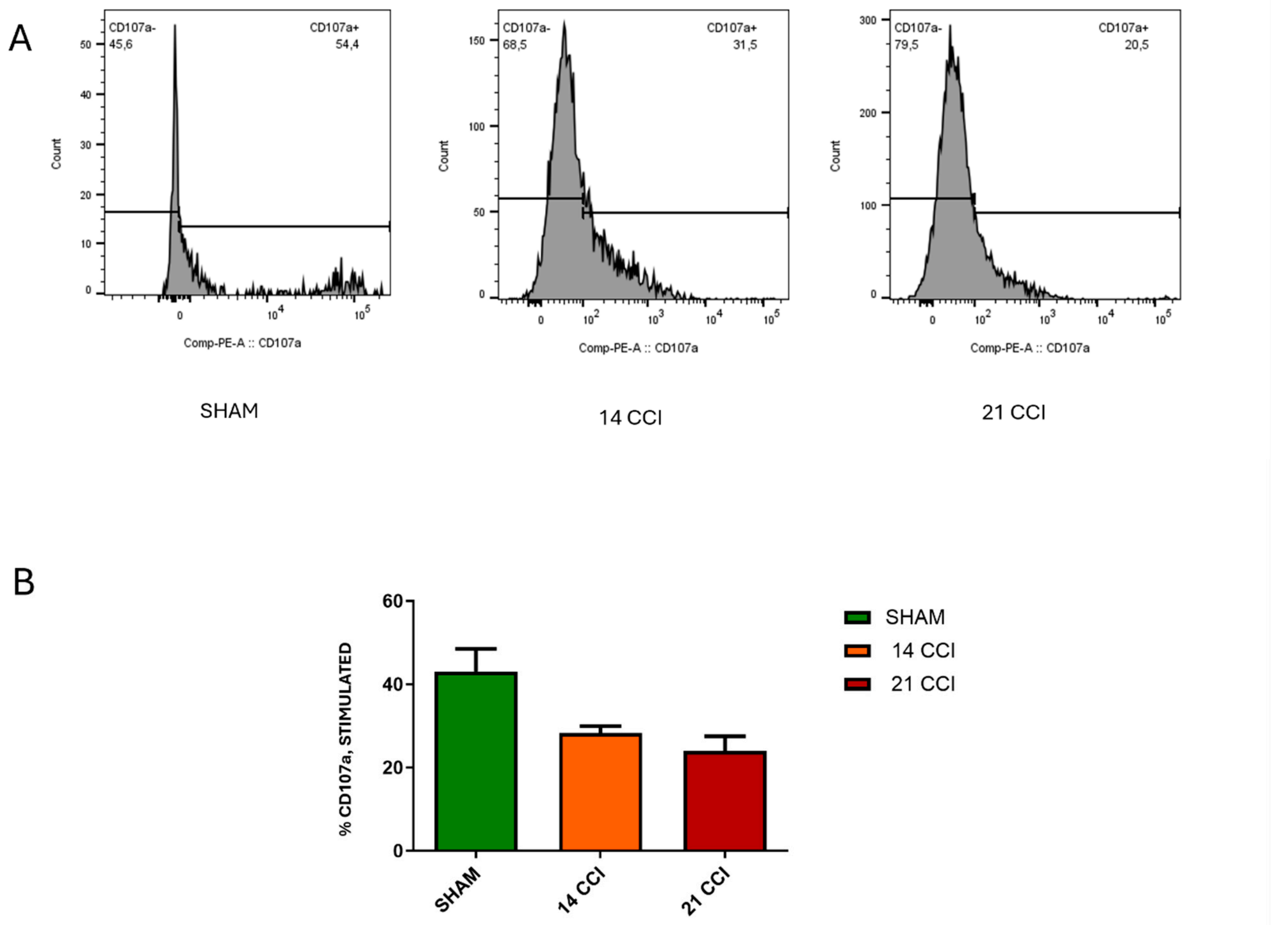

Figure S2

*Events were gated on live singlet lymphocytes, CD3⁻NK1.1⁺ NK cells, and further gated to exclude cells expressing Ly49A. CD107 cell surface expression on Ly49C/I at 14 and 21 days after CCI (C and D); PMA/Ionomycin was added to positive controls. Results are expressed as mean ± SEM for 3 different experiments. * p value< 0.05*

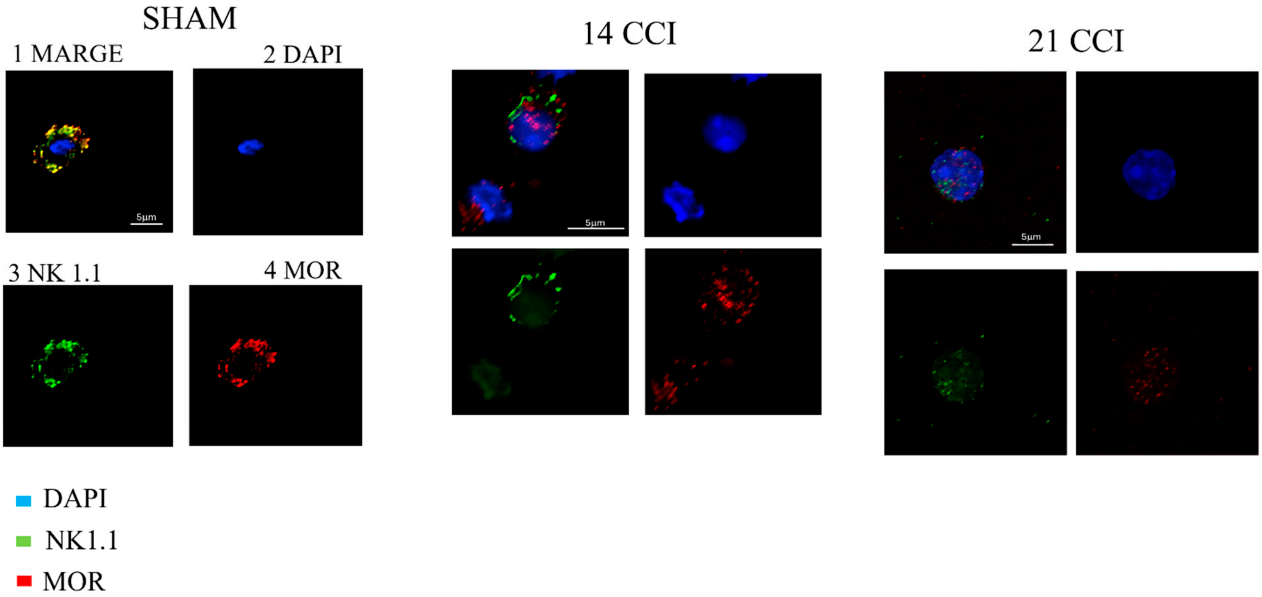

Figure S3

Confocal microscopy analysis of the granule pattern in NK cells from sham B6 mice at ×60 magnification: SHAM, MARGE (Figure 1), DAPI (blue) nucleus (Figure 2), NK.1.1 (green) to NK cells marker (Figure 3); MOR (red) indicating receptor localization (Figure4), for sham mice (A), CCI group for 14 days (B), CCI for 21 days (C). Scale bar= 5 μm.

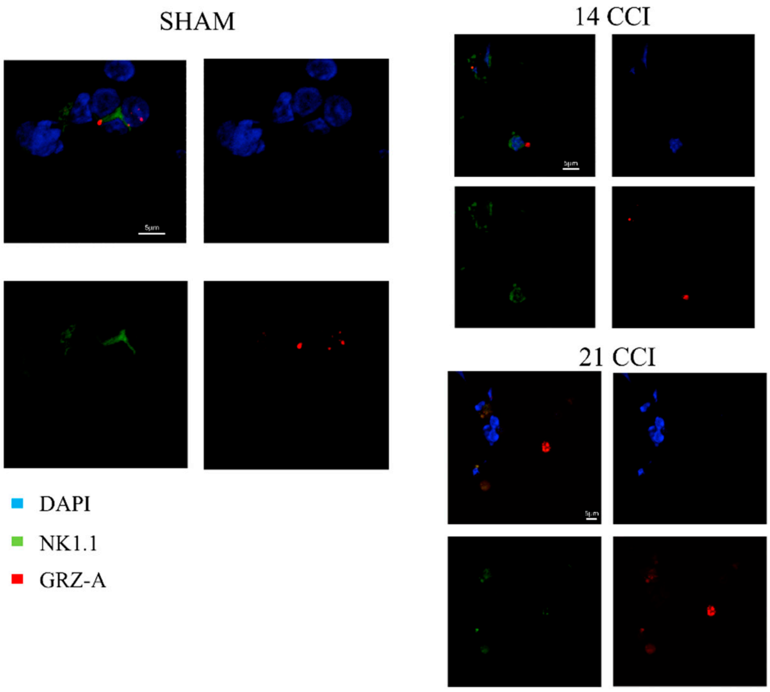

Figure S4

Confocal microscopy analysis of the granule pattern in NK cells from sham B6 mice, CTRL, MARGE (Figure 1). DAPI (blue) nucleus (Figure 2), NK.1.1 (green) to NK cells marker (Figure 3); GRZ-A (red) indicating granules localization (Figure 4), for sham mice (A), CCI group for 14 days (B), CCI for 21 days (C). Scale bar= 5 μm.

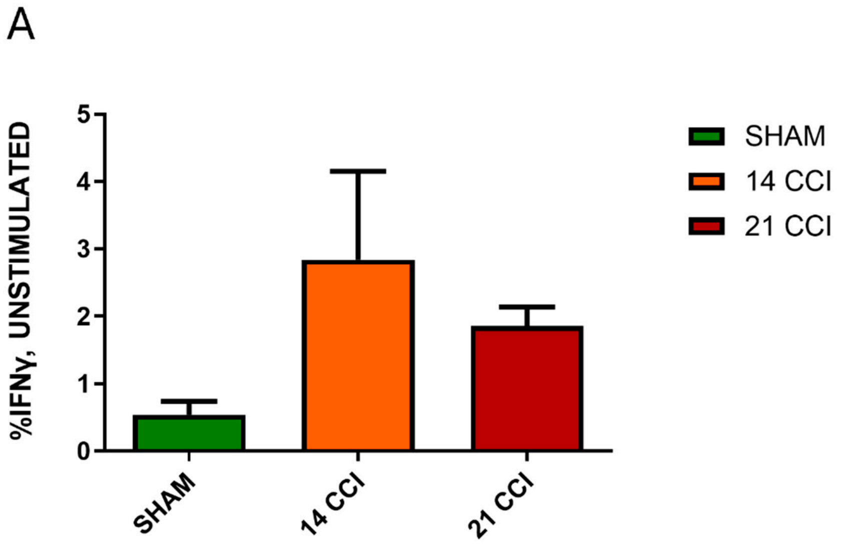

Figure S5A

*Flow cytometry analysis for measuring production of IFNγ, under stimulated conditions with PMA//I. Events were gated on live singlet lymphocytes, CD3−NK1.1+ NK cells at 14 and 21 days after CCI. Unstimulated conditions. Results are expressed as mean ± SEM for 3 different experiments *p value<0.0050.*

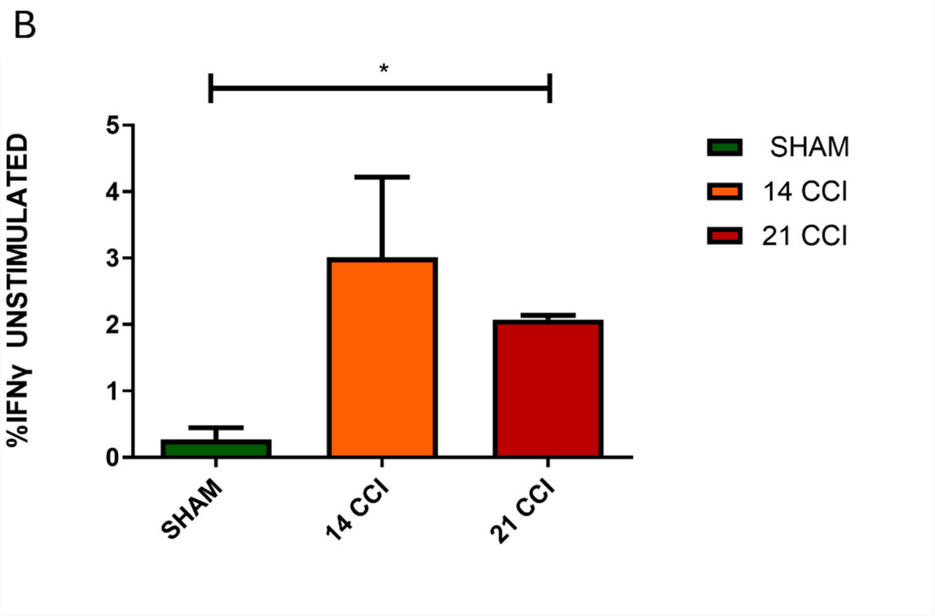

Figure S5B

*Flow cytometry analysis for measuring cytokine release in B6 mice at 14 and 21 days after CCI. Lymphocytes were incubated for 2-3 h at 37°C. We performed surface staining followed by intracellular staining in the presence of an interferon antibody. Events were gated on live singlet lymphocytes, CD3−NK1.1+ NK cells, and further gated to exclude cells expressing Ly49A. Intracellular expression of IFNγ on the Ly49C/I gate. Unstimulated conditions. Results are expressed as mean ± SEM for 3 different experiments *p value <0.050.*
